# Adequacy of Anesthesia Guidance for Combined General/Epidural Anesthesia in Patients Undergoing Open Abdominal Infrarenal Aortic Aneurysm Repair; Preliminary Report on Hemodynamic Stability and Pain Perception

**DOI:** 10.3390/ph17111497

**Published:** 2024-11-07

**Authors:** Michał Jan Stasiowski, Seweryn Król, Paweł Wodecki, Nikola Zmarzły, Beniamin Oskar Grabarek

**Affiliations:** 1 Chair and Department of Emergency Medicine, Faculty of Medical Sciences in Zabrze, Medical University of Silesia, 40-760 Katowice, Poland; 2 Department of Anaesthesiology and Intensive Care, 5th Regional Hospital, 41-200 Sosnowiec, Poland; seweryn.krol@gmail.com; 3 Department of General, Colorectal and Polytrauma Surgery, Faculty of Health Sciences in Katowice, Medical University of Silesia, 40-555 Katowice, Poland; 4 Department of Vascular Surgery, 5th Regional Hospital, 41-200 Sosnowiec, Poland; hydrops@onet.pl; 5 Collegium Medicum, WSB University, 41-300 Dabrowa Gornicza, Poland; nikola.zmarzly@wsb.edu.pl (N.Z.); bgrabarek7@gmail.com (B.O.G.)

**Keywords:** Adequacy of Anaesthesia, epidural anesthesia, ropivacaine, bupivacaine, surgical pleth index, open lumbar infrarenal aortic aneurysm repair

## Abstract

**Background/Objectives**: Hemodynamic instability and inappropriate postoperative pain perception (IPPP) with their consequences constitute an anesthesiological challenge in patients undergoing primary elective open lumbar infrarenal aortic aneurysm repair (OLIAAR) under general anesthesia (GA), as suboptimal administration of intravenous rescue opioid analgesics (IROAs), whose titration is optimized by Adequacy of Anaesthesia (AoA) guidance, constitutes a risk of adverse events. Intravenous or thoracic epidural anesthesia (TEA) techniques of preventive analgesia have been added to GA to minimize these adverse events. **Methods**: Seventy-five patients undergoing OLIAAR were randomly assigned to receive TEA with 0.2% ropivacaine (RPV) with fentanyl (FNT) 2.5 μg/mL (RPV group) or 0.2% bupivacaine (BPV) with FNT 2.5 μg/mL (BPV group) or intravenous metamizole/tramadol (MT group). IROA using FNT during GA was administered under AoA guidance. Systemic morphine was administered as a rescue agent in all groups postoperatively in the case of IPPP, assessed using the Numeric Pain Rating Score > 3. The maximum score at admission and the minimum at discharge from the postoperative care unit to the Department of Vascular Surgery, perioperative hemodynamic stability, and demand for rescue opioid analgesia were analyzed. **Results**: Ultimately, 57 patients were analyzed. In 49% of patients undergoing OLIAAR, preventive analgesia did not prevent the incidence of IPPP, which was not statistically significant between groups. No case of acute postoperative pain perception was noted in the RPV group, but at the cost of statistically significant minimum mean arterial pressure values, reflecting hemodynamic instability, with clinical significance < 65mmHg. Demand for postoperative morphine was not statistically significantly different between groups, contrary to significantly lower doses of IROA using FNT in patients receiving TEA. **Conclusions**: AoA guidance for IROA administration with FNT blunted the preventive analgesia effect of TEA compared with intravenous MT that ensured proper perioperative hemodynamic stability along with adequate postoperative pain control with acceptable demand for postoperative morphine.

## 1. Introduction

Open major abdominal surgery is one of the riskiest surgical procedures performed under GA for IPPP and hemodynamic instability, while TEA still constitutes the gold standard analgesic regimen for the upper abdomen [1] as it was proven to provide improved postoperative analgesia and reduce the incidence of chronic postoperative pain compared with parenteral opioids [2,3]. Therefore, it should always be considered as a routine adjunct to GA in elective OLIAAR [4].

Monitors of analgesia quality that measure nociception/anti-nociception balance, the intensity of nociception (painful stimulation) and the efficacy of anti-nociception (pain relief), are increasingly gaining popularity [5]. The AoA concept is based on monitoring the depth of GA detected by a forehead sensor using the entropy electroencephalogram (response entropy, RE; state entropy, SE) and the surgical pleth index (SPI) obtained from the finger photoplethysmography signal, both of which do not require complex preoperative preparations [6]. The observation of SE values within the range of 40–60 as a result of the proper administration of the hypnotic GA component, reflecting the correct suppression of the limbic system, together with the observation of the increase in the SPI value on the monitor (0—no painful stimulation, 100—maximal painful stimulation) after the painful stimulus and a return to the baseline level after an intravenous bolus (anti-nociception) of rescue opioid analgesia (IROA), makes monitoring with AoA guidance easy [7]. SPI has been successfully used to monitor intra- and postoperative analgesia and also helps guide IROA [8]. When SPI monitoring was employed, fewer adverse events, reduced opioid consumption, faster emergence from GA [9,10], and less postoperative pain [11] were reported.

Considering all of the above, we designed a randomized controlled study to assess the effect of TEA using a combination of 0.2% ropivacaine (RPV) fentanyl (FNT) or 0.2% bupivacaine (BPV) and FNT on intra- and postoperative demand for opioids and hemodynamic stability compared with intravenous preventive analgesia using metamizole/tramadol (MT) in patients undergoing OLIAAR under AoA-guided GA.

## 2. Results

**Figure 1:**
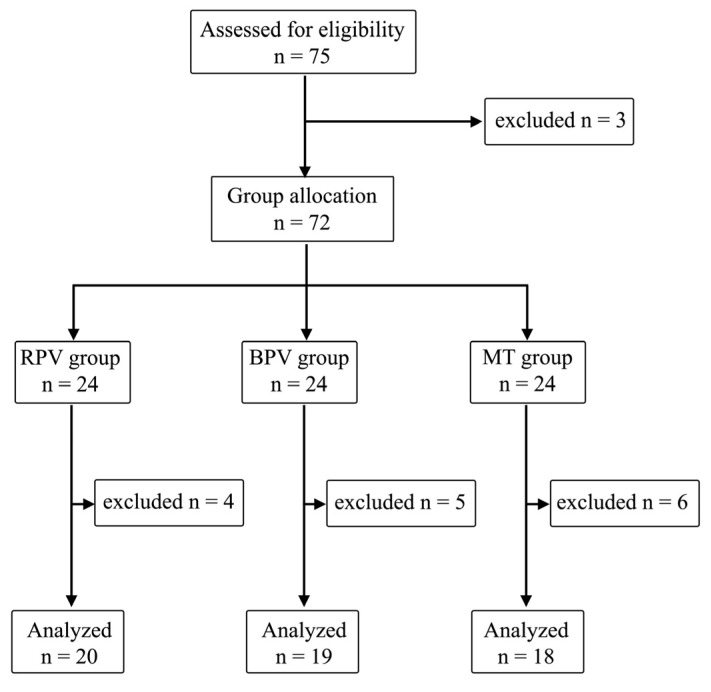
Randomization graph. n, number of cases.

Seventy-five patients were enrolled for this study. After assignment for eligibility, one patient withdrew previous consent, and two patients were disqualified from OLIAAR due to surgical reasons. After the 72 patients were assigned to three equal groups, 15 patients were excluded from final analysis: 5 patients (3 from the BPV group and 2 from the RPV group) were disqualified due to failed thoracic epidural catheter placement, 3 patients due to intraoperative shock requiring temporary intravenous infusion of catecholamines impairing SPI monitoring, according to the manufacturers of AoA monitoring, 2 patients due to intraoperative heart rhythm disturbances impairing SPI monitoring and requiring intraoperative administration of pharmacological antiarrhythmic drugs, 3 patients due to intraoperative peripheral hypothermia caused by massive fluid challenge impairing SPI monitoring, 2 patients due to change in surgical technique as a result of impaired blood flow after declamping of the aorta requiring peripheral aortofemoral bypass. The final analysis included 57 patients, 10 women (17.5%) and 47 men (82.5%), randomly assigned to three groups (Figure 1).

Detailed characteristics of the anthropometric data of the patients are shown in Table 1. There were no significant differences between the groups in the case of age, height, weight, and BMI.

Detailed characteristics of the incidence of intraoperative interventions in patients overall and according to group allocation are presented in Table 2.

No significant differences between the groups regarding OLIAAR time, number of patients requiring IROA administration using FNT, volume of intraoperative fluid therapy, demand for intraoperative red blood cell transfusion (concentrate of red blood cells plus cell saver), and number of patients requiring intraoperative administration of rescue atropine, ephedrine, and urapidil, including the demand for their intraoperative rescue doses, in contrast to the need for IROA, which was statistically significantly lower in the RPV and BPV group compared to the MT group.

Detailed characteristics of postoperative pain experienced by patients and the frequency of postoperative interventions depending on group allocation are shown in Table 3.

No significant differences between groups regarding time of OLIAAR, maximum and minimum NPRS values, frequency of the first postoperative main perceptions: acute, moderate, mild, and IPPP (Supplementary Figure S1), and the number of patients requiring postoperative rescue morphine in the Post-Anesthesia Care Unit were registered, although the demand for intraoperative dose of IROA using FNT was higher in the MT group compared to both RPV and BPV groups, as reported in the previous table (Table 2).

Detailed characteristics of the mean values of perioperative parameters in patients overall and according to group allocation are shown in Table 4.

No significant hemodynamic changes were noted in Stage 1 and Stage 2 regarding the mean values of SAP, MAP, DAP, HR, SE, and SPI between the studied groups. The mean SAP, MAP, and DAP values were significantly higher in the MT group compared to the RPV group and BPV group during Stage 3. In Stage 4, mean SAP was significantly higher in the MT group compared to the RPV and BPV groups.

Detailed characteristics of the maximum and minimum values of perioperative parameters in patients overall and according to group allocation are shown in Table 5.

No significant hemodynamic changes were noted in Stage 2 regarding the maximum and minimum values of SAP, MAP, DAP, HR, SE, and SPI between the studied groups. During Stage 3, the minimum values of SAP, MAP, and DAP were significantly higher in the MT group compared to the RPV group and between the MT group and both the BPV and RPV groups in terms of the minimum MAP values as the most important hemodynamic parameter expressing the intraoperative safety of patients (Supplementary Figure S2). Similarly, in Stage 4, the minimum SAP, MAP, and DAP values were higher in the MT group compared to the RPV and BPV groups.

## 3. Discussion

The current study analysis covering the utility of preventive analgesia in patients undergoing OLIAAR revealed the incidence of IPPP in 28 of 57 patients (49%) despite group allocation. In the group of patients receiving TEA using 0.2% RPV with FNT 2.5 μg/mL, only 6 of 20 patients reported postoperative pain other than mild, whereas receiving TEA using 0.2% BPV with FNT 2.5 μg/mL resulted in IPPP in 10 of 19 patients, and preventive analgesia using metamizole with tramadol resulted in IPPP in 12 of 18 patients, which did not appear to be statistically significant (*p* = 0.07). Interestingly, no cases of acute postoperative pain were reported in the RPV group, contrary to the other two groups, where such observations were noted in six patients in each. Despite the group allocation, the demand for postoperative morphine in the early postoperative period in the Post-Anesthesia Care Unit was 7.9 ± 5.3 mg. In the RPV group, the demand for postoperative morphine was 4 ± 3.1 mg, whereas it was 7.7 ± 4.8 mg in the BPV group and 10 ± 5.7 mg in the preemptive MT group, which was not statistically significant (*p* = 0.1).

In ASA III patients with cardiovascular comorbidities, IPPP constitutes a serious hazard because it activates the stress hormone release, which may lead to the decompensation of atherosclerotic plaques and result in cardio-vasculo-cerebral incidents in the perioperative period [12]. An adequate comprehensive approach to pain management minimizes the overall consumption of analgesics, increases postoperative patient mobility, shortens hospital stay, optimizes multifactorial patient outcomes, and optimizes resources. Therefore, an anesthetic regimen is sought to ensure proper perioperative analgesia in order to take countermeasures against the development of the abovementioned serious adverse events. On the one hand, proper intraoperative monitoring of nociception/anti-nociception balance to guide IROA ensures the prevention of central sensitization to diminish IPPP [13], while on the other hand, successful preventive analgesia using either intravenous medication or TEA has been proven to provide an adequate reduction in the incidence of IPPP in patients undergoing OLIAR [14]. In this study, we decided on a novel approach to employ both AoA guidance of IROA along with preventive analgesia, intravenous or TEA, using either BPV or RPV. RPV is a long-acting amide local anesthetic that has been synthesized to reversibly bind and inactivate sodium channels in the open state. It possesses the potential to inhibit sodium ion influx in nerve fibers and block the propagation of action potentials. Compared to BPV, it is less likely to penetrate large myelinated motor fibers and acts selectively on A, B, and C nociceptive fibers. Being manufactured as the pure S(-) enantiomer, it is characterized by significantly lower cardiotoxicity and neurotoxicity, as RPV isomers have been proven to possess lesser cardiodepressant effects than BPV isomers due to the replacement of the butyl group with a propyl-terminal group [15].

The incidence of IPPP may be associated with increased morbidity, has been proven to affect the quality of life and impair recovery, and constitutes a risk factor for the development of persistent pain and long-term opioid use [16]. Although all medications have side effects, opioids have particularly concerning multisystemic long-term and short-term side effects that may increase morbidity. Therefore, the concept of perioperative multimodal analgesia or preventive analgesia was designed to combine reduced minimally effective doses of analgesics that act on different sites and pathways in an additive or synergistic manner, leading to pain relief with minimal or even no opiate consumption (opioid-free anesthesia) [17]. Preventive analgesia has been shown to reduce the intensity of postoperative pain perception using regional [18], epidural [19], or intravenous techniques using tramadol [20,21] or cyclooxygenase inhibitors [22,23,24,25,26,27], although there are conflicting data regarding the influence of their use on the IROA sparring effect and the reduction in adverse events related to IROA dosage, similarly to tramadol. Some studies also confirm the superiority of TEA over parenteral opioids in reducing the intensity of IPPP in patients undergoing OLIAAR [28,29,30]. Although there are conflicting data concerning the superiority of TEA over alternative analgesic techniques used as part of an enhanced recovery protocol in terms of the incidences of desaturation, quality of recovery, or risk of postoperative morbidity [31,32], this may be due to the fact that the failure rate of TEA is higher than generally recognized, reaching up to 40% [33].

Since variations in SPI values in response to nociceptive stimulation have been proven to correlate with serum opioid concentration [34], the employment of AoA guidance for IROA has optimized its requirement during GA [10] and has proven useful in reducing the intensity of IPPP expressed by NPRS compared with standard practice in patients undergoing different surgical procedures [35]. Nociception/anti-nociception monitoring devices seem to have an advantage over standard clinical practice for intraoperative management of analgesia during GA [36]. Jain et al. [13] compared the utility of SPI guidance for FNT administration with the standard practice based on observation of hemodynamic changes and reported that higher doses of IROA using FNT were required with lower postoperative rescue analgesic requirements, which indirectly revealed that conventional anesthetic regimens lead to intraoperative underdosing of IROA, resulting in the development of IPPP in the mechanism of central sensitization. Similarly, Won et al. observed that in elderly patients, who constitute the majority of the patients in this study, the AoA guidance for analgesia provided appropriate analgesia with sufficient intraoperative remifentanil consumption, a lower incidence of hypertension/tachycardia events, and a lower incidence of delirium in the Post-Anesthesia Care Unit than the conventional analgesia [37], which supports the thesis of a direct effect of AoA guidance on IROA administration leading to a reduction in perioperative adverse events. More importantly, a growing number of reports confirm the utility of observing changes in AoA values at certain stages of surgery in predicting IPPP [38,39,40,41,42], which allows anesthesiologists to modify the intraoperative anesthesiological regimen according to the individual needs of each patient undergoing specific surgical procedures in order to minimize the risk of IPPP before extubation.

Proper intraoperative IROA administration under AoA guidance has also been shown to contribute to intraoperative stability, reducing the incidence of hypertension/tachycardia events more than the conventional guidance of intraoperative analgesia [37]. It has been proven that improper intraoperative blood pressure management can lead to hypoperfusion of vital organs associated with hypotension [43], as intraoperative hypotension constitutes a frequent and significant health risk to their function in the perioperative period and can impair and prolong recovery [44]. Proper intraoperative fluid therapy has become a cornerstone of perioperative management, as it can significantly influence the treatment outcome [45]. Therefore, the fundamental goal of intraoperative fluid therapy is to maintain physiological fluid and electrolyte balance to avoid excessive loading of water, sodium, and chloride [46,47]. Policies are directed to predict major adverse cardiovascular events after noncardiac surgeries [48,49], associated with elevated troponin [49] and myocardial infarction at 30 days [50]. Increased mortality [51], stroke [52], acute kidney injury [53], and delirium [54] were also reported following intraoperative hypotension incidences. Intraoperative hypotension was defined as decreases in MAP values ≤ 65 mmHg [55], and in the present study, the intraoperative minimum MAP values in both the BPV and RPV groups met the criteria for intraoperative hypotension (stage 3), compared with the MT group, where the minimum MAP value did not meet the above criteria and was higher than 65mmHg, which appeared to be of statistical significance (see Table 4) and of great clinical relevance. Since the volume of intraoperative fluid therapy, the demand for intraoperative red blood cell transfusion (red blood cell concentrate plus cell saver), and the number of patients requiring intraoperative rescue administration of atropine, ephedrine, and urapidil, including the demand for their intraoperative rescue doses (see Table 2) did not statistically significantly differ between groups, the only factor that could be responsible for mean values of MAP < 65 mmHg during stage 3—OLIAAR surgery—is TEA, regardless of the local anesthetics used.

In terms of intraoperative fluid therapy, there has been a debate concerning the superiority of a restrictive versus liberal regimen for maintaining hemodynamic stability. In the study of Warrillow et al. [56], intraoperative fluid challenge during major gastrointestinal surgery was analyzed. In their study, the mean dose (± SD) of fluids was 4229 ± 1840 mL (in total), 3762 ± 1687 mL (for crystalloids), and 467 ± 601 mL (for colloids), which was similar to the demand in the current study despite the group allocation, although intraoperative blood loss during major vascular surgery is known to be higher than that during gastrointestinal surgery. On the other hand, Jia et al. proved that patients receiving a “restrictive” intraoperative fluid challenge model enjoyed a shorter hospital stay and faster recovery, although no improvement in survival was observed [57]. Nevertheless, although the presence of intraoperative hypotension is known to be relevant for postoperative organ disfunction, fortunately, adverse events like postoperative renal disfunction, ischemic myocardial infarction, or ischemic stroke were not observed in the current study [29], probably as a side effect of the appropriate fluid challenge that we chose instead of a restrictive regimen to meet the vascular surgery team’s expectations of good preload of aortic prosthesis under the condition of preoperative myocardial ischemia resulting in a mildly impaired left ventricular ejection fraction, but higher than 40%, supposedly occurring in the vast majority of ASA III patients undergoing OLIAAR.

As patients who died in the retrospective study by Czajka et al. received significantly more fluids than survivors, in the present study, we adopted a multimodal approach combining liberal fluid challenge with maintaining a hemoglobin concentration > 10g% intraoperatively by the employment of autotransfusion of red blood cells using a cell saver as first-line therapy and red blood cell transfusion [58].

Some authors have recently proven that intraoperative norepinephrine infusion is a promising solution to intraoperative hypotension [59], proving its safety in stabilizing the intraoperative MAP [60,61]. Abovementioned studies, especially that published by Aykanat et al., concern surgeries with mean blood loss around 250 milliliters and intraoperative fluid challenge around 1600 milliliters [59], and in no case was epidural analgesia employed, while both local anesthetics and norepinephrine may induce cardiac arrhythmia separately [62], not mentioning the fact that norepinephrine was reported to not so significantly increase the systemic vascular resistance following spinal anesthesia [63] Moreover, norepinephrine infusion was long regarded as a contraindication to IROA guidance under AoA, even by its manufacturer, and only one study has been published so far regarding AoA reliability under episodes of bleeding and norepinephrine infusion in patients undergoing craniotomy for the evacuation of subdural or extradural hematoma [64], long after the current study was designed and started, which also requires further studies, in our experience, especially in ASA III patients with general atherosclerosis and its complications, like heart failure, which is very common in patients undergoing OLIAAR, when, to say the least, incidences of intraoperative bleeding are incomparable to those observed during neurosurgical procedures.

In summary, it should be emphasized that in the RPV group, we observed a trend towards a reduced incidence of acute postoperative pain perception compared with the two other groups, as no incidence of acute postoperative pain was observed in patients receiving TEA using RPV with FNT. On the other hand, unfortunately, this was achieved at the expense of hemodynamic safety, as lower minimum MAP values were reported in patients receiving TEA compared with the MT group, supposedly as a result of peripheral vasodilatation [65] or a toxic influence on the myocardium via direct negative chronotropic, dromotropic, and inotropic effects impairing myocardial contractility [66] or both. Pharmacokinetic differences in the lipophilicity of local anesthetics correlate well with the depression of mitochondrial ATP synthesis in fast-metabolizing myocardial cells. The lack of difference in hemodynamic stability parameters between patients allocated to the BPV and RPV groups is surprising because RPV is manufactured as the pure S(-) enantiomer, which is characterized by significantly less cardiotoxicity and neurotoxicity [15]. RPV isomers were advertised by their manufacturers as having less cardiodepressant effects than BPV isomers due to the replacement of the butyl group with a propyl-terminal group, which surprisingly was not the case in this study as bupivacaine was reported to produce a significantly greater depression of ejection fraction of left ventricle than ropivacaine, mepivacaine, or lidocaine [66]. Our observations are consistent with those of Dai et al., who identified intrathecal anesthesia as an independent risk factor for intraoperative hypoperfusion, among other patient characteristics in the current study, such as older age, high ASA grade, physical status, and history of hypertension [67]. They also emphasize the appropriate treatment of intraoperative hypotension during surgery, which is consistent with our methodology of maintaining hemoglobin levels above >10 mg% using a cell saver and transfusion of red blood cell concentrate, rather than focusing on hemodynamic monitoring of left ventricular ejection fraction and systemic vascular resistance, which may not be widely available and, in patients with general atherosclerosis, may show different values depending on the catheterized artery, which may lead the anesthesiologist to make wrong decisions when accompanied with incorrect SPI values during temporary arterial bleeding in the course of aortic declamping after proximal and distal anastomosis.

Since TEA is not free from potential adverse effects or harms [68], such as spinal hematoma, cauda equina syndrome, meningitis, and epidural abscess [69], not to mention the numerous contraindications to its use, further studies should be carried out to investigate the utility of AoA guidance in patients receiving GA with preventive analgesia using metamizole and tramadol, possibly with regional anesthesia techniques such as ultrasound-guided bilateral rectus sheath block or erector spinae plane block, possibly using RPV with adjuvants as the most effective in the current study to combine effective analgesia with hemodynamic safety in the quickly growing population of elderly patients undergoing OLIAR, similar to the suggestion made by Ragavendran et al. [19] in their study on pain perception after aortofemoral bypass or wound infiltration, followed by continuous infusion of local anesthetics plus a single intravenous bolus of morphine, which was proven to provide similar quality of postoperative analgesia in the study of Ball et al. to continuous TEA in patients undergoing OLIAAR [70], which we are currently trying to introduce in our center as a promising compromise between efficient pain management and hemodynamic stability. The only benefit of TEA, regardless of the local anesthetics used, was a statistically significant reduction in the mean demand of IROA using FNT compared with the MT group, which is based on our anesthesiological experience and intuition derived from our previous projects regarding the quality of postoperative infiltration anesthesia [71] or postoperative analgesia after dual guidance regional blocks [18], using RPV in both cases as the most modern LA, in this case, cannot outweigh the potential risk of neurological deficits, which fortunately were not observed in this study. If regional anesthesia techniques of preventive analgesia also fail to provide effective pain control compared with intravenous PA, we suggest that the risk of potential perioperative complications associated with the administration of local anesthetics compared with intravenous analgesia outweigh the potential benefits when IROA is administered intraoperatively under AoA guidance. We made a similar observation in our recently published study concerning vitreoretinal surgeries, where the addition of peribulbar block, despite the local anesthetics mixture used, did not prove superior over intravenous paracetamol in a single dose of 1 g, when GA was performed using AoA guidance for IROA administration, analyzing postoperative pain perception and perioperative hemodynamic stability [72], when a cardiodepressive effect of even small doses of local anesthetics was also observed. Therefore, it is advisable to make a one-time investment in modern AoA monitoring rather than risk unnecessary complications in every patient in daily practice, with the accompanying legal consequences.

This study has several notable advantages and limitations. One key advantage is the comprehensive approach to analgesia, utilizing both TEA and intravenous preventive analgesia to assess different pain management strategies in patients undergoing OLIAAR. The use of AoA monitoring for IROA administration is another strength, allowing precise opioid titration and enhancing intraoperative hemodynamic stability, particularly in patients with cardiovascular comorbidities. AoA guidance contributed to reduced postoperative morphine demand in TEA groups, though this result was not statistically significant. However, this study faced limitations, including the risk of intraoperative hypotension in the TEA groups, which may pose a clinical hazard. We adopted a protocol where the indication for the administration of IROA was an ∆SPI of >15; however, this was in accordance with previous studies concerning the utility of the AoA guidance for GA in patients undergoing different surgical procedures [73]. We intended to avoid miscalculations in the case of low values of SPI and the possible overdosing of IROA. However, the employment of a stricter protocol, even though the standards in the current literature report that an ∆SPI of >10 or an SPI of >50 constitutes an indication for the administration of IROA, could have possibly resulted in hazardous opioid-induced bradycardia and hypotension, thereby potentially harming the patient [74], possibly magnifying the potential hypotensive effect of TEA.

Furthermore, a notable number of patients experienced failed epidural catheter placements, reducing the sample size for final analysis and highlighting technical challenges, which are widely reported as one of the main disadvantages of epidural analgesia, reaching up to 24.8% of cases [2,75,76]. The relatively small overall sample size (57 patients) limits the statistical power to detect significant differences between groups. Additionally, this study did not include a control group without preventive analgesia, which could have provided further insights into the isolated effects of AoA-guided IROA. Finally, there were potential biases in AoA readings during episodes of cardiac arrhythmia or bleeding, potentially leading to inappropriate opioid administration. These factors should be considered when interpreting the findings, as they balance this study’s strengths with important limitations.

The relationships between postoperative hemodynamic parameters and pain perception using PHHPS to indirectly assess respiratory effects, NPRS and SPI values, and PONV incidence rate will be analyzed separately due to a word count limit, similar to our previous studies on the utility of AoA guidance [41]. The perception of postoperative pain is a subjective phenomenon that is difficult to quantify [77]. We did not deliberately analyze the postoperative pain intensity after discharge from the Post-Anesthesia Care Unit to the Department of Vascular Surgery because the project involved monitoring of NPRS as well as SPI values in Stage 4, while patient arousal (changing bed position, coughing, etc.) markedly interferes with SPI monitoring [39]; therefore, such comparison has no clinical value [78], and we focused on short-term outcomes, similarly to the study of Salata et al. [4]. We intentionally did not study the group without PA, because some authors claim that titration of IROA under SPI guidance did not show any clinical value in reducing the incidence of IPPP, so such a methodology could become bioethically questionable from their perspective [10,79]. We also did not study combined TEA/GA groups without AoA guidance because such studies have been conducted and the results are known [29], and titration of IROA under nociception/anti-nociception balance monitoring using different methods has become a worldwide standard in clinical studies [7]. Finally, out of 72 patients, 15 of them were disqualified from the final analysis; therefore, it must be underlined that both TEA and SPI monitoring are challenging and not always applicable. Therefore, it must always be taken into consideration that an anesthesiologist must be aware of the potential necessity of employment of standard monitoring during OLIAAR, as the AoA regimen has its limitations, especially during episodes of cardiac arrhythmia or bleeding with hypotension, when hemodynamic centralization leads to impairment of peripheral blood flow, showing high SPI values, being an indication for the administration of IROA, possibly worsening the hypotension caused by bleeding, when blindly following AoA indices, not correlating with patient’s condition at that time. Similar attention must be paid to erroneous values of RE and SE, which may lead inexperienced anesthesiologists to make a wrong decision, possibly leading to either administering a toxic dose of a hypnotic drug, inducing hypotension with all potential consequences, or intraoperative awareness with recall [80].

## 4. Materials and Methods

### 4.1. Patients

Patients scheduled for elective OLIAAR in the Department of Vascular Surgery of St. Barbara Memorial 5th Regional Hospital in Sosnowiec, Poland, and meeting the inclusion criteria were asked to participate in the parallel, prospective, randomized clinical trial and prepared for anesthesia and surgery according to the criteria included in contemporary guidelines for cardiovascular assessment and management of patients undergoing non-cardiac surgery issued by the European Society of Cardiology. Seventy-five patients with American Society of Anaesthesiologists (ASA) score III were enrolled after providing written informed consent. Randomization was performed by opening sealed envelopes by a third author. In compliance with the Helsinki Declaration, ethical approval for this study (KNW/0022/KB1/125/17) was provided by the Bioethical Committee of the Medical University of Silesia on 5 December 2017 (Chairman Ph. Dr. Bogusław Okopień). The project was registered in the Clinical Trial Registry (Silesian MUKOAiIT11, NCT0660999), and the data were collected from 5 December 2017, to 31 March 2020, when the data collection was halted a result of the COVID-19 pandemic with the first lockdown, when the members of the research team were transferred to the intensive care unit for SARS-CoV-2 patients.

Exclusion criteria included history of allergy to local anesthetics, metamizole, or tramadol, pre-existing cardiovascular disease (cardiac arrhythmia, antiplatelet therapy), and patients at risk of intraoperative hypotension (low left ventricle ejection fraction < 40%), who may require excessive fluid resuscitation or administration of vasoactive drugs that may interfere with SPI monitoring.

All patients were familiarized with the 10-point Numeric Pain Rating Scale (NPRS; 0 indicated no pain and 10 indicated worst imaginable pain) during the pre-anesthesia consultation the day before the planned surgery to assess pain intensity and preventive analgesia techniques. All study participants were monitored for 48 h concerning the evaluation of adverse events. The score was recorded every 10 min in the Post-Anesthesia Care Unit.

In the BPV group, upon arrival to the operating room, patients received AoA-guided GA combined with preventive analgesia using an epidural mixture of 0.2% BPV (Bupivacainum Hydrochloricum WZF 0.5%, 5 mg/mL, 10 mL, Polfa Warszawa S.A, Warsaw, Poland) with FNT 2.5 μg/mL, administered 20 min before induction of GA, because the addition of FNT to the epidural analgesic mixture proved superior to local anesthetics alone, as shown by Khanna et al. [81].

In the RPV group, upon arrival to the operating room, patients received AoA-guided GA combined with preventive analgesia using an epidural mixture of 0.2% RPV (Ropivacaini Hydrochloridum 1%, 10 mg/mL, 10 mL, Molteni Farmaceutici, Italy) with FNT 2.5 μg/mL, administered 20 min before induction of GA.

In the MT group, upon arrival to the operating room, patients received AoA-guided GA combined with intravenous preventive analgesia in the form of a single dose of 16mg/kg metamizole (Pyralgin 0.5g/mL, 2 mL solution; Polpharma, Poland) and 100 mg tramadol (Tramadoli hydrochloricum, 50 mg/mL solution; Polpharma, Poland), both in 100 mL of saline solution, 30 min before arrival to the operating room.

All patients fasted for at least 12 h due to potential diabetic gastropathy. On the day of surgery, before the start of anesthesia, they received medication at a dose of 3.75–7.5 mg/kg, depending on age and body weight [82].

Immediately before the procedure, patients were pre-oxygenated for 5 min with 100% oxygen and intravenously administered Ringer’s solution at a dose of 10–15 mL/kg body weight. Anesthesia was induced intravenously with FNT at a dose of 2 mcg/kg body weight and a single dose of 0.3 mg/kg of body weight etomidate (Etomidate Lipuro, 2 mg/mL, 10 mL, Braun, Germany) administered intravenously at a dose of 0.2-0.3 mg/kg body weight to achieve SE of approximately 40–45 [80].

All patients were paralyzed with a standard intravenous dose of 0.6 mg/kg rocuronium (Esmeron, Fresenius, Poland) after loss of consciousness. After 70 s, oral intubation was performed. CO_2_ was maintained at 35–37 mmHg. Before the start of OLIAAR surgery, the sevoflurane level was maintained at approximately 40–45 SE, similarly to our previous projects on intravenous preventive analgesia in vitreoretinal surgeries [35,83], underlining the cardioprotective properties of sevoflurane, dedicated to ASA III patients [84,85].

Standard monitoring procedures were used during induction of anesthesia and OLIAAR, with particular attention paid to such vital parameters as heart rate (HR), noninvasive arterial pressure (NIBP), standard electrocardiography (ECG) II, arterial blood saturation (SaO_2_), exhaled carbon dioxide concentration (etCO_2_), fraction of inspired oxygen in the gas mixture (FiO_2_), minimal alveolar concentration of sevoflurane (MAC), fraction of inspired sevoflurane (FiAA), and fraction of expired sevoflurane (FeAA).

An entropic electroencephalogram (SE and RE) was used to monitor the depth of anesthesia, intraoperative analgesia was guided with the surgical pleth index (SPI), and NMT monitoring (Carescape B650, GE, Helsinki, Finland) was used to maintain muscle relaxation.

### 4.2. Stage 1

The electroencephalogram entropy (RE, SE) sensor on the patient’s forehead, the pulse oximeter (SPI) on the contralateral finger to the venous access, the NIBP cuff on the right arm, and the standard 5-lead ECG on the patient’s back were placed after admission to the operating theatre according to manufacturer’s recommendations, and then the first values were recorded.

#### TEA Technique

The procedure was performed in the sitting position to identify the epidural space using ultrasound imagining. Ultrasonographic visualization was carried out using Sonosite equipped with a convex transducer operating at a frequency of 13 MHz. The paravertebral region of the thoracic vertebral column was scanned starting from the lateral side of the spinous processes of the 7th and 8th thoracic vertebrae to identify the pleura and ribs (characteristic visualization called “saw teeth”), the transverse processes of the upper vertebrae (characteristic visualization called “camels’ humps”), and the epidural space (characteristic visualization called “batman’s head”). A non-sterile, hypoallergenic transmission gel was used. After skin disinfection and surgical, sterile draping of the planned injection site, the patient’s skin was infiltrated with 2 mL of lidocaine solution. A sterile cover was placed over the transducer. Sterile transmission gel was used during the procedure. The distance from the skin surface to the epidural space was measured to introduce the needle at proper depth using an extended epidural set (extended epidural set, Balton, Łajski, Poland) employing an ultrasound-assisted technique until no resistance was achieved in a disposable low-resistance syringe; alternatively, the technique of observing the conversion of the convex into concave water droplet shape at the end of Tuohy needle could be used. For TEA, an 18 G epidural catheter was inserted into the epidural space 3 to 5 cm cranially via an 18 G, 100 mm Tuohy needle with an entry point between the T7 and T8 vertebrae. The catheter was covered with a transparent epi-fix dressing (Unomedical, Poznań, Poland). To rule out intravascular injection or subarachnoid or subdural block, 4 mL of a test solution of 2% lidocaine with epinephrine (Lidocaine HCl 2%, 40 mg/200 mL (20 mg/dL) and epinephrine 1:200,000, injection USP, Hospira Inc., Lake Forest, IL, USA) was administered. To detect any sensory block, a cold swab test was performed 20 min prior to epidural administration of the single-shot bolus of the anesthetic mixture, according to group allocation.

The initial bolus volume of the anesthetic mixture was calculated according to the Bromage equation (0.8 mL + 0.05 mL/per each 5 cm of height > 150 cm to block one spinal segment) to ensure sensory block (12 spinal segments from the 7th thoracic to the 2nd lumbar) of the abdomen wall, similar to the technique described in a study by Steinberg et al. [86] and the official in-hospital preventive analgesia procedure dedicated to abdominal surgery. Then, after positioning the patient horizontally on the operating table, preventive analgesia was followed by a continuous infusion of 0.1 mL/kg body weight of epidural analgesic mixture per hour for up to 72 h, similar to the technique described in the study by Capdevila et al. [87] or a continuous infusion of metamizole to a maximum daily dose of 5 g and tramadol to maximum daily dose of 0.6 g.

### 4.3. Stage 2

In Stage 2, to calculate the mean SPI value, SPI values starting at 5 min after tracheal intubation were recorded until sterilization of the surgical site was initiated, allowing calibration of the SPI sensor. We observed whether an initial FNT dose of 2 mcg/kg body weight provided sufficient analgesia, as reflected by SPI < 40 and 0.8 minimal alveolar concentration of sevoflurane, with the adequate depth of hypnosis reflected by SE 40–45. Otherwise, an additional FNT dose of 1 mcg/kg body weight was added, and the minimal alveolar concentration of sevoflurane was similarly increased by 0.1 to reach proper values. Gruenewald et al. [88] proposed ∆SPI > 10 or an absolute SPI value > 50 as a predictor of inadequate analgesia. On the other hand, in other studies, only an absolute value of ∆SPI > 50 was an indication for rescue analgesia [89].

If mean arterial pressure (MAP) was lower than 65 mmHg, supposedly due to postinduction hypotension [55] exacerbated by TEA, resulting mainly in a reduction in peripheral vascular resistance [90] or, less likely, in disruption of either cardiac output by cardiotoxic local anesthetics [91] absorbed from the epidural space into the central vascular system or by cardiodepressant hypnotics and opioid analgesics, additional boluses of intravenous crystalloids were infused in a volume of 5% Ringer’s solution at a dose of 5 mL/kg body weight until MAP was restored to > 65 mmHg.

### 4.4. Stage 3—OLIAAR

All OLIAAR surgeries, according to the group assignment, were performed by the same specialist general and vascular surgeon (P.W—second author) with more than 15 years of on-site and abroad experience in this field. SE and SPI values were monitored online, and the values were recorded at a frequency of 1 min. Achieving ∆SPI > 15 points above the mean SPI value of Stage 2 resulted in the administration of IROA 1 mcg/kg body weight FNT intravenously every 5 min until the SPI value reached the mean SPI value of Stage 2. The duration of OLIAAR was counted from the first incision to the last suture placement, including the following stages: skin incision, opening of the abdomen, opening of the retroperitoneal space, preparation of the aortic aneurysm sac, aortic cross-clamping, prosthesis implantation, declamping of the aorta, closure of the retroperitoneal space, closure of the abdomen, and skin sutures, for which the mean values of all analyzed parameters will be analyzed separately and published to assess the quality of PA, similarly to our previous project analyzing similar aspects of preventive analgesia quality at different stages of vitreoretinal surgeries [92].

To avoid a potentially dangerous overdose of FNT due to the potential miscalculation of the SPI score due to its variations, this study adopted a compromise protocol of ∆SPI > 15 compared to the calculated baseline value during Stage 2 as an indicator of demand for IROA, similarly to all our recently published and cited studies on the utility of AoA guidance in the field of colonoscopy [73], endoscopic sinus surgery [93], lumbar discectomy [71], and vitreoretinal surgery [35,83].

Patients received an infusion of 10 mL/kg/h of balanced crystalloids, while synthetic colloids were transfused as rescue therapy solely to restore intravascular volume resulting from current blood loss at a dose of 5 mL/kg body weight per incidence of each equal blood loss [94]. Intraoperatively, noninvasive blood pressure monitoring was used in accordance with fluid therapy guided by metabolic equilibrium and maintenance of hemoglobin concentration > 10 g% [58], in standard situations measured after aortic declamping, when patients were administered 40 milliequivalents of 8.4% natrium bicarbonate (Natrium bicarbonicum 8.4% Polpharma Warszawa, Poland) to restore hemodynamic stability due to reperfusion syndrome, as disturbances in cardiac output or systemic vascular resistance can be induced by various patient- and procedure-related factors, including bleeding, drug-mediated vasodilation, or cardiac depression [95].

### 4.5. Stage 4—Post-Anesthesia Care Unit

After the emergence from GA and extubation, all patients were further monitored (SPI, HR, SAP, MAP, DAP, SaO_2_) in the Post-Anesthesia Care Unit by different anesthesia teams (second author). In the case of IPPP in patients receiving TEA, the quality of the block was assessed; an additional bolus of the analgesic mixture was added when a sensory deficiency of the block was diagnosed, using the appropriate volume calculated based on the Bromage scale to block deficient dermatomes. Despite the group allocation, if a patient declared IPPP, they were titrated with a 2 mg morphine bolus (Morphini Sulfas WZF, 20mg/mL, solutio pro iniectione, Polfa Warszawa, Poland) every 10 min until an NPRS value < 4 was declared. In addition to postoperative hemodynamic parameters, each patient was monitored for adverse events such as early postoperative nausea and vomiting (early PONV in the Post-Anesthesia Care Unit and late PONV in the Department of Vascular Surgery), sedation level, and allergic reactions, along with pain assessment, for 24 h. Monitoring and data recording were discontinued, except for PONV cases, which were recorded in the postoperative period within the first 48 h, and in each case, intravenous antiemetics were administered: first-line therapy with Ondansetron (Ondansetron Accord 2 mg/mL, 2 mL solution, Accord Healthcare Limited, Great Britain) and rescue therapy with Dexaven (Dexaven 4 mg/mL, Jelfa, Poland), both at a dose of 4 mg.

In the case of MAP < 65 mmHg, Optylite solution was administered at a dose of 5 mL/kg body weight. Patients received oxygen at a rate of 3 L/min via a nasal cannula. Every 10 min, patients were asked about their pain intensity perception based on the Numeric Pain Rating Scale (NPRS), ranging from 0 (no pain) to 10 (maximum pain) and PHHPS ranging from 0 (no pain at cough) to 4 (severe pain at rest). In the case of NPRS > 3, a standard dose of nonsteroidal anti-inflammatory drugs or metamizole was administered according to the patient’s individual needs and the contemporary guidelines for acute pain treatment issued by the Polish Society of Anaesthesiologists in 2014 with a further update in 2018 [96,97]. SPI values were monitored online, and mean values were recorded every 1 min (trends in software provided by the producer). NPRS, PHHPS, and SPI values were recorded for different pain perception ranges: mild pain (NPRS 0–3), moderate pain (NPRS 4–6), acute pain (NPRS 7–10), no pain at cough (PHHPS 0), pain at cough but not with deep breathing (PHHPS 1), pain at deep breathing but not at rest (PHHPS 2), slight pain at rest (PHHPS 3), severe pain at rest (PHHPS 4). Before patients were transferred from the Post-Anesthesia Care Unit to the Department of Vascular Surgery, they were monitored for at least 30 min. Monitoring and data recording were discontinued, except for PONV cases, which were recorded in the postoperative period within the first 48 h.

All patients were discharged to the Department of Vascular Surgery when they met 4 conditions: postoperative pain perception at rest NPRS < 4, PHHPS < 2, MAP > 65 mmHg, HR > 60 or <90 beats/minute, Aldrete score > 8.

### 4.6. Statistical Analysis

Statistical analysis was carried out with STATISTICA 13.3 (StatSoft, Kraków, Poland). The Shapiro–Wilk test was used to confirm the lack of normality in data distribution. The Kruskal–Wallis test and Dunn’s post hoc test were performed. Mean, standard deviation, median, and interquartile range were shown for quantitative data. Qualitative data were analyzed with the chi-square test and presented with percentages. Statistical significance was set at the level *p* < 0.05.

The group size was calculated using G*Power 3.1.9.7 [98]. One-way ANOVA (f = 0.4, α = 0.05, power = 0.8) for 3 groups estimated the total sample size of 66. As data from 57 of the 75 patients were analyzed, a post hoc test was carried out and revealed that with this sample size, the power was 0.75.

## 5. Conclusions

This study yielded both positive results and identified notable adverse effects, requiring a balanced interpretation. On the positive side, the use of TEA with RPV and BPV demonstrated a trend toward reduced postoperative pain perception, particularly in the RPV group, where no cases of acute postoperative pain were reported. This suggests that TEA, when guided by AoA monitoring, offers effective analgesia and decreases the need for postoperative opioids, as shown by the lower demand for morphine in TEA groups compared to the intravenous analgesia group.

However, these benefits came with notable adverse effects. The TEA groups, particularly those receiving RPV, experienced significant intraoperative hypotension, reflected in lower minimum MAP values compared to the intravenous analgesia group. This suggests that while TEA effectively manages pain, it also increases the risk of hemodynamic instability, likely due to peripheral vasodilation or direct myocardial depression caused by local anesthetics. The AoA monitoring system, though beneficial in optimizing opioid administration, posed challenges during episodes of cardiac arrhythmia or bleeding. In such instances, the system’s reliance on peripheral blood flow led to potentially misleading indices, which could result in inappropriate opioid administration and worsen hypotension.

In summary, while TEA demonstrated clear advantages in reducing postoperative pain and opioid requirements, these benefits must be weighed against the increased risk of intraoperative hypotension. Anesthesiologists must be vigilant in balancing pain control with hemodynamic safety, particularly when using AoA monitoring, to ensure optimal patient outcomes.

## Figures and Tables

**Table 1:** Anthropometric data of patients in total and by group allocation.

Data		Total N = 57 (100%)	RPV Group N = 20 (35%)	BPV Group N = 19 (33%)	MT Group N = 18 (32%)	*p*-Value
Gender N (%)	female	10 (17.5%)	5 (25%)	3 (15.8%)	2 (11.1%)	0.5 NS
	male	47 (82.5%)	15 (75%)	16 (84.2%)	16 (88.9%)	
Age X ± SD Me (IQR)	years	66.6 ± 6.7 66 (9)	66.6 ± 6.8 67 (11)	66.5 ± 7.1 66 (8)	66.8 ± 6.4 66 (8.5)	1.0 NS
Height X ± SD Me (IQR)	centimeters	172.3 ± 6.2 174 (6)	171.8 ± 6.5 174 (11)	171.5 ± 7 173 (7)	173.7 ± 4.8 174 (6)	0.7 NS
Weight X ± SD Me (IQR)	kilograms	77.8 ± 13.6 80 (17)	76.9 ±11 81 (20)	79.4 ± 18.3 82 (26)	77.4 ± 11.8 78 (15)	0.8 NS
BMI X ± SD Me (IQR)	kilograms/meter^2^	26.2 ± 4.1 25.8 (4.6)	26.1 ± 3.4 25.6 (4.5)	26.8 ± 5.1 26 (7.2)	25.7 ± 4 25.8 (4.1)	0.7 NS
DM N (%)	yes	11 (19.3%)	3 (15%)	3 (15.8%)	5 (27.8%)	0.5 NS

RPV—ropivacaine group; BPV—bupivacaine group; MT—metamizole/tramadol group; SD—standard deviation; Me—median; IQR—interquartile range; BMI—body mass index; DM—diabetes mellitus; NS—not significant.

**Table 2:** Rate of incidence of intraoperative interventions.

Parameter		Total N = 57 (100%)	RPV Group N = 20 (35%)	BPV Group N = 19 (33%)	MT Group N = 18 (32%)	*p*-Value
Time of OLIAAR X ± SD Me (IQR)	min	124.1 ± 44.8 113 (41)	137.2 ± 44.4 131.5 (39.5)	116.6 ± 51.5 108 (62)	117.6 ± 35.8 108 (44)	0.3 NS
Number of patients requiring IROA administration using FNT	N (%)	46 (81%)	15 (75%)	14 (74%)	17 (94%)	0.2 NS
Intraoperative need for IROA administration using FNT X ± SD Me (IQR)	mcg	196.5 ± 162.5 200 (200)	137.5 ± 115.7 125 (150)	147.4 ± 125.2 100 (300)	313.9 ± 184.6 300 (250)	RPV vs. MT, *p* = 0.07 BPV vs. MT, *p* = 0.02
Intraoperative fluid therapy volume X ± SD Me (IQR)	mL	4251 ± 925.3 4500 (1340)	4208.2 ± 852.4 4540 (1000)	4341.5 ± 1053.9 4500 (1500)	4220 ± 949.2 4320 (1040)	0.8 NS
Demand for intraoperative red blood cell transfusion (red blood cell concentrate plus cell saver) X ± SD Me (IQR)	mL	671 ± 671.3 477 (544)	630.4 ± 659.6 454 (332)	620.8 ± 593.8 500 (420)	766.8 ± 784.2 462.5 (1048)	0.9 NS
Number of patients requiring intraoperative rescue atropine	N (%)	13 (23%)	3 (15%)	6 (32%)	4 (22%)	0.5 NS
Demand for intraoperative dose of rescue atropine X ± SD Me (IQR)	mcg	569.2 ± 154.8 500 (0)	533.3 ± 57.7 500 (100)	633.3 ± 216 500 (300)	500 ± 0 500 (0)	0.4 NS
Number of patients requiring intraoperative rescue ephedrine	N (%)	29 (51%)	12 (60%)	10 (53%)	7 (39%)	0.5 NS
Demand for intraoperative dose of rescue ephedrine X ± SD Me (IQR)	mg	25.6 ± 14.4 25 (10)	25.2 ± 13.8 25 (3.6)	25.8 ± 13.9 25 (10)	24.6 ± 17.8 15 (22.5)	0.9 NS
Number of patients requiring intraoperative rescue urapidil	N (%)	10 (18%)	3 (15%)	3 (16%)	4 (22%)	0.8 NS
Demand for intraoperative dose of rescue urapidil X ± SD Me (IQR)	mg	16.3 ± 13.2 10 (10)	13.3 ± 5.8 10 (10)	24.2 ± 22.4 12.5 (40)	12.5 ± 8.7 10 (10)	0.5 NS

RPV—ropivacaine group; BPV—bupivacaine group; MT—metamizole/tramadol group; OLIAAR— open lumbar infrarenal aortic aneurysm repair; IROA— intraoperative rescue opioid analgesia; FNT—fentanyl; mcg—microgram; mg—milligram; SD—standard deviation; Me—median; IQR—interquartile range; NS—not significant.

**Table 3:** Postoperative pain perception and incidence of postoperative interventions depending on group allocation.

Data		Total N = 57 (100%)	RPV Group N = 20 (35%)	BPV Group N = 19 (33%)	MT Group N = 18 (32%)	*p*-Value
NPRS max X ± SD Me (IQR)	[1 ÷ 10]	3.2 ± 3.3 3 (5.5)	2 ± 2.4 0 (5)	3.2 ± 3.5 2.5 (7)	4.4 ± 3.6 5 (7)	0.1 NS
NPRS min X ± SD Me (IQR)	[1 ÷ 10]	1.7 ± 1.9 0 (3)	1.2 ± 1.6 0 (3)	1.6 ± 2.1 0 (3)	2.4 ± 1.9 3 (4)	0.1 NS
Type of first postoperative pain perception N (%)	mild	29 (51%)	14 (70%)	9 (47%)	6 (33%)	0.07 NS
	moderate	16 (28%)	6 (30%)	4 (21%)	6 (33%)	0.7 NS
	acute	12 (21%)	0 (0%)	6 (32%)	6 (33%)	1.0 NS
	IPPP	28 (49%)	6 (30%)	10 (52%)	12 (67%)	0.7 NS
PHHPS during mild pain perception	[1 ÷ 4]	0.8 ± 0.6 1 (1)	0.9 ± 0.4 1 (0)	0.7 ± 0.6 1 (1)	0.8 ± 0.9 1 (1)	0.5 NS
PHHPS during moderate pain perception	[1 ÷ 4]	2.6 ± 0.6 3 (1)	2.6 ± 0.5 3 (1)	2.7 ± 0.5 3 (1)	2.5 ± 0.7 3 (1)	0.8 NS
PHHPS during acute pain perception	[1 ÷ 4]	3.6 ± 0.7 4 (1)	-	3.6 ± 0.5 4 (1)	3.7 ± 0.8 4 (0)	1.0 NS
Number of patients requiring postoperative rescue morphine in the PACU	N (%)	28 (49%)	6 (30%)	10 (52%)	12 (67%)	0.7 NS
Dose of postoperative morphine required in the PACU X ± SD Me (IQR)	mg	7.9 ± 5.3 8 (8)	4 ± 3.1 3 (2)	7.7 ± 4.8 8 (8)	10 ± 5.7 10 (9)	0.1 NS

RPV—ropivacaine; BPV—bupivacaine; MT—metamizole/tramadol; NPRS—Numeric Pain Rating Score; IPPP—inappropriate postoperative pain perception; PHHPS—Prince Henry Hospital Pain Score; PACU—postoperative care unit; mg—milligram; SD—standard deviation; Me—median; IQR—interquartile range; NS—not significant.

**Table 4:** Mean values of perioperative parameters.

Parameter X ± SD Me (IQR)	Total N = 57 (100%)	RPV Group N = 20 (35%)	BPV Group N = 19 (33%)	MT Group N = 18 (32%)	*p*-Value
Stage 1					
HR (beats/min)	72.1 ± 12.9 69 (15)	75 ± 10.3 74.5 (13.5)	70.9 ± 16.9 67 (24)	69.9 ± 10.5 67 (10)	0.2 NS
SAP (mmHg)	151.7 ± 24 154 (37)	153.7 ± 25.5 158 (38)	153.2 ± 23.1 154 (47)	148.1 ± 24.2 150 (38)	0.8 NS
MAP (mmHg)	108 ±14.2 110 (22)	109.2 ± 17.4 117 (24.5)	108.6 ± 11.6 111 (20)	106.2 ± 13.4 107 (18)	0.5 NS
DAP (mmHg)	78.5 ± 10.5 77 (15)	80.4 ± 12.8 80.5 (15)	76.9 ± 8.4 76 (10)	78.3 ± 9.9 80.5 (16)	0.6 NS
SE	87.8 ± 7.2 89 (1.5)	89.7 ± 1.2 90 (1.5)	87.8 ± 4.4 89 (3)	85.6 ± 12 89 (5)	0.08 NS
SPI	61.6 ± 16.4 66 (23)	63 ± 18.6 70 (30)	60.3 ± 18.6 66 (24)	61.4 ± 11.4 64 (18)	0.7 NS
Stage 2					
mean HR (beats/min)	66.7 ± 12.7 64.8 (16.7)	68.4 ± 9.6 69.4 (14.8)	69.5 ± 16.7 68.5 (20.6)	61.7 ± 9.7 60 (12.8)	0.09 NS
mean SAP (mmHg)	116 ± 21.9 111 (27.5)	119.6 ± 23.1 113.8 (39.2)	121.2 ± 23.1 116.5 (36.5)	106.4 ±16.7 104.5 (24)	0.1 NS
mean MAP (mmHg)	84.9 ± 14.9 82 (17)	89.2 ± 17.2 86.3 (21.1)	87.2 ± 14.1 83.5 (19)	77.6 ± 10.5 78.2 (14)	0.05 NS
mean DAP (mmHg)	64 ± 11.3 64 (13.3)	65.1 ± 8.1 65.4 (11.3)	67.8 ± 14.9 64 (21.3)	58.8 ± 8.3 57.7 (13)	0.05 NS
mean SE	46.7 ± 7.4 48.3 (7.8)	46.4 ± 9.1 47 (8.8)	46.1 ± 6.9 46.4 (8.3)	47.8 ± 6 49.6 (4.7)	0.8 NS
mean SPI	33.4 ± 13.7 31.1 (16.4)	35.8 ± 11.7 32.4 (12.2)	35.9 ± 16.7 30.3 (23.5)	28.1 ± 11.1 26.8 (17.2)	0.2 NS
mean FiAA	2.2 ± 3.5 1.7 (0.3)	3 ± 5.8 1.7 (0.9)	1.7 ± 0.2 1.7 (0.2)	1.8 ± 0.3 1.8 (0.2)	0.4 NS
mean FeAA	1.4 ± 0.3 1.3 (0.4)	1.4 ± 0.4 1.4 (0.6)	1.3 ± 0.2 1.2 (0.3)	1.4 ± 0.3 1.4 (0.2)	0.2 NS
mean MAC	0.7 ± 0.1 0.7 (0.2)	0.7 ± 0.2 0.7 (0.3)	0.7 ± 0.1 0.6 (0.2)	0.7 ± 0.1 0.7 (0.1)	0.2 NS
Stage 3—OLIAAR					
mean HR (beats/min)	65.4 ± 9.6 63 (11.7)	66 ± 8 64.3 (10.5)	65.5 ± 12.1 62 (8.3)	64.7 ± 8.8 63.4 (16.8)	0.5 NS
mean SAP (mmHg)	114.6 ± 18.7 113.8 (21.2)	107.5 ± 12.9 106.1 (18.4)	108.6 ± 19.2 106 (33.7)	128.9 ± 16.2 123 (29.1)	RPV vs. MT, *p* = 0.001 BPV vs. MT, *p* = 0.004
mean MAP (mmHg)	83.9 ± 11.4 83.7 (15.7)	79.8 ± 8.5 80 (12.2)	79.8 ± 11.5 79.4 (20.3)	93 ± 9.3 90.3 (17.1)	RPV vs. MT, *p* = 0.001 BPV vs. MT, *p* = 0.002
mean DAP (mmHg)	63.3 ± 9.3 63.8 (11.2)	60.1 ± 8.1 60.8 (9.8)	60.5 ± 9 62.6 (12.3)	69.8 ± 7.6 67.3 (10.4)	RPV vs. MT, *p* = 0.002 BPV vs. MT, *p* = 0.007
mean SE	43.5 ± 7.2 43.9 (7.7)	44.5 ± 7.8 44.1 (4.1)	44.2 ± 6.6 45.2 (9.4)	41.6 ± 7.2 40.5 (10.8)	0.4 NS
mean SPI	40.3 ± 11.6 40.5 (18.6)	42.3 ± 11.5 43.4 (20.8)	37.4 ± 10.8 35.9 (14.2)	41.2 ± 12.5 41.7 (15.5)	0.3 NS
mean FiAA	1.5 ± 0.3 1.5 (0.4)	1.6 ± 0.3 1.6 (0.4)	1.5 ± 0.3 1.5 (0.5)	1.6 ± 0.3 1.5 (0.4)	0.6 NS
mean FeAA	1.3 ±0.2 1.3 (0.3)	1.3 ± 0.2 1.4 (0.4)	1.3 ± 0.3 1.4 (0.4)	1.3 ± 0.2 1.3 (0.3)	0.9 NS
mean MAC	0.7 ± 0.2 0.7 (0.2)	0.7 ± 0.1 0.7 (0.2)	0.7 ± 0.3 0.6 (0.2)	0.7 ± 0.1 0.7 (0.2)	0.3 NS
Stage 4—PACU					
mean HR (beats/min)	70.5 ± 14.9 68.9 (17.7)	70.4 ± 12 69.9 (18.3)	69.2 ± 16.9 67.5 (24)	71.9 ± 16.1 68.1 (17.7)	0.8 NS
mean SAP (mmHg)	130.9 ± 22.9 128.3 (27.9)	125.7 ± 19.8 124.7 (29.3)	123 ± 22.1 127 (31.3)	145.1 ± 21.5 144.1 (24)	RPV vs. MT, *p* = 0.03 BPV vs. MT, *p* = 0.01
mean MAP (mmHg)	94.2 ± 13.7 93.5 (14.9)	92.4 ± 14.3 89.5 (16.6)	90 ± 13.1 92.5 (18.4)	100.5 ± 12.2 99.6 (13.4)	0.05 NS
mean DAP (mmHg)	68.6 ± 11.1 68.3 (14)	66 ± 11.7 67.4 (15.5)	67.2 ± 10.9 68 (14.4)	72.9 ± 10 71.8 (10.3)	0.1 NS
mean SPI	56.9 ± 12.3 54.7 (15.9)	54.5 ± 9.1 53 (14.9)	55.1 ± 14.4 54.4 (14.2)	61.6 ± 12.3 62.8 (20.7)	0.2 NS

RPV—ropivacaine; BPV—bupivacaine; MT—metamizole/tramadol; HR—heart rate; SAP—systolic arterial pressure; MAP—mean arterial pressure; DAP—diastolic arterial pressure; SE—state entropy; SPI—surgical pleth index; FiAA—fraction of inspired sevoflurane; FeAA—fraction of expired sevoflurane; MAC—minimal alveolar concentration of sevoflurane; OLIAAR—open lumbar infrarenal aortic aneurysm repair; PACU—Post-Anesthesia Care Unit; SD—standard deviation; Me—median; IQR—interquartile range; NS—not significant.

**Table 5:** Fluctuations in perioperative parameter values.

Parameter X ± SD Me (IQR)	Total N = 57 (100%)	RPV Group N = 20 (35%)	BPV Group N = 19 (33%)	MT Group N = 18 (32%)	*p*-Value
Stage 2					
max HR (beats/min)	79.4 ± 15.1 80 (23)	82 ± 14.9 81 (24.5)	83 ± 15.5 82 (22)	72.7 ± 13.3 73 (20)	0.1 NS
max SAP (mmHg)	135 ± 25.3 134 (37)	138.8 ± 28.5 137.5 (45.5)	137.1 ± 24.2 141 (38)	128.8 ± 22.9 127 (26)	0.4 NS
max MAP (mmHg)	96.8 ± 15.3 93 (22)	99.8 ± 16 103 (26.5)	98.2 ± 17.1 99 (21)	92.1 ± 11.9 92.5 (14)	0.2 NS
max DAP (mmHg)	74.1 ± 13.1 72 (16)	74.7 ± 11.5 77 (19.5)	76.7 ± 17.6 73 (19)	70.8 ± 8.4 71 (7)	0.6 NS
max SE	55 ± 11.2 55 (12)	54.7 ± 10.2 56 (30)	53.6 ± 11.1 54 (16)	56.9 ± 12.7 54 (6)	0.8 NS
max SPI	53.9 ± 17.9 53 (27)	59.5 ± 18.8 59 (30)	55.1 ± 16.1 54 (25)	46.6 ± 17.1 47.5 (6)	0.1 NS
max FiAA	2 ± 0.4 1.9 (0.5)	2 ± 0.4 2.1 (0.7)	1.9 ± 0.3 1.9 (0.3)	2 ± 0.3 1.9 (0.2)	0.4 NS
max FeAA	1.5 ± 0.3 1.5 (0.5)	1.6 ± 0.4 1.7 (0.7)	1.5 ± 0.3 1.5 (0.4)	1.6 ± 0.2 1.5 (0.2)	0.4 NS
max MAC	0.8 ± 0.1 0.8 (0.2)	0.8 ± 0.2 0.9 (0.4)	0.8 ± 0.1 0.7 (0.1)	0.8 ± 0.1 0.8 (0.2)	0.3 NS
min HR (beats/min)	59.9 ± 12.1 59 (14.3)	60.4 ± 9.2 59.5 (10)	63.9 ± 15.8 62 (42)	55.1 ± 9 52.5 (13)	0.1 NS
min SAP (mmHg)	101.6 ± 22.4 99 (31)	103.5 ± 19 100.5 (26.5)	109 ± 25.9 104 (42)	91.7 ± 19.1 91.5 (30)	0.1 NS
min MAP (mmHg)	74.1 ± 13.6 75 (17)	76.2 ± 11.6 76.5 (17)	78 ± 14.7 77 (28)	67.8 ± 13 68.5 (17)	0.1 NS
min DAP (mmHg)	55.9 ± 11.9 56 (14)	56.7 ± 9 57 (13.5)	60.5 ± 13.6 58 (25)	50.3 ± 11.2 48.5 (15)	0.1 NS
min SE	38.9 ± 9.1 40 (10)	38.4 ± 10.2 42 (9)	38.1 ± 9.1 38 (9)	40.7 ± 8 41 (11)	0.5 NS
min SPI	20.4 ± 11.8 17 (11)	19.5 ± 6.7 18.5 (12)	23.9 ± 16.5 17 (20)	17.8 ± 10 14.5 (11)	0.5 NS
min FiAA	1.6 ± 0.4 1.6 (0.5)	1.6 ± 0.5 1.7 (0.9)	1.5 ± 0.3 1.5 (0.3)	1.7 ± 0.4 1.6 (0.3)	0.4 NS
min FeAA	1.2 ± 0.3 1.2 (0.4)	1.2 ± 0.4 1.3 (0.7)	1.1 ± 0.2 1.1 (0.3)	1.3 ± 0.3 1.3 (0.5)	0.1 NS
min MAC	0.6 ± 0.2 0.6 (0.2)	0.6 ± 0.2 0.7 (0.3)	0.5 ± 0.1 0.6 (0.3)	0.7 ± 0.1 0.7 (0.1)	0.1 NS
Stage 3—OLIAAR					
max HR (beats/min)	84.4 ± 15.5 82 (19)	86.4 ± 15.6 83 (21.5)	85.5 ± 18.2 78 (16)	80.9 ± 12.1 82.5 (19)	0.6 NS
max SAP (mmHg)	154.4 ± 28.3 152 (40)	146.5 ± 25.1 147 (48)	148.1 ± 27.2 150 (43)	169.8 ± 27.8 169 (42)	0.05 NS
max MAP (mmHg)	110.7 ± 18.1 109 (23)	106.4 ± 16 107.5 (25.5)	107.1 ± 17 104 (21)	119.3 ± 19.2 115.5 (28)	0.1 NS
max DAP (mmHg)	85.4 ± 14.6 84 (17)	82.5 ± 13.7 85 (17)	83.7 ± 15 79 (19)	90.3 ± 14.7 86 (20)	0.4 NS
max SE	58.7 ± 12.9 56 (12.5)	60.1 ± 12.5 56 (11.5)	58.6 ± 11.6 56 (11)	57.2 ± 15 54 (15)	0.5 NS
max SPI	73.1 ± 13.8 76 (16)	74.3 ± 15.3 78.5 (12)	72.3 ± 10.7 76 (10)	72.7 ± 15.7 75 (25)	0.5 NS
max FiAA	2 ± 0.4 2 (0.4)	2.1 ± 0.3 2.1 (0.4)	1.9 ± 0.4 2 (0.5)	2.1 ± 0.4 2 (0.4)	0.1 NS
max FeAA	1.7 ± 0.3 1.7 (0.4)	1.7 ± 0.2 1.7 (0.3)	1.6 ± 0.3 1.6 (0.5)	1.7 ± 0.3 1.7 (0.5)	0.4 NS
max MAC	0.9 ± 0.2 0.9 (0.2)	0.9 ± 0.3 0.9 (0.2)	0.9 ± 0.3 0.8 (0.3)	0.9 ± 0.2 0.9 (0.2)	0.6 NS
min HR (beats/min)	53.6 ± 9.7 53 (10)	54 ± 6.3 53.5 (9)	54.5 ± 13.6 54 (11)	52.4 ± 8 50 (12)	0.6 NS
min SAP (mmHg)	82.7 ± 17.4 80 (19)	75.6 ± 10.9 77 (18.5)	80.9 ± 17.2 80 (17)	92.6 ± 19.6 91 (30)	RPV vs. MT, *p* = 0.01
min MAP (mmHg)	62.2 ± 11.4 61 (15)	57.9 ± 8.2 58 (9)	60.1 ± 11.7 58 (13)	69.4 ± 11.4 69 (14)	RPV vs. MT, *p* = 0.008 BPV vs. MT, *p* = 0.03
min DAP (mmHg)	47.3 ± 8.5 46 (10)	44.3 ± 6.5 44.5 (7)	45.6 ± 8.2 46 (13)	52.4 ± 8.8 51.5 (10)	RPV vs. MT, *p* = 0.008
min SE	32.4 ± 8.3 32 (14)	32 ± 8.9 31 (15)	33.2 ± 7.7 37 (13)	32.1 ± 8.5 33 (6)	0.8 NS
min SPI	14.7 ± 7.8 13 (8)	15.8 ± 6.1 15.5 (9)	13.2 ± 9.7 11 (6)	15.1 ± 7.3 12.5 (12)	0.2 NS
min FiAA	1.2 ± 0.3 1.2 (0.4)	1.2 ± 0.3 1.2 (0.4)	1.2 ± 0.3 1.1 (0.6)	1.2 ± 0.4 1.2 (0.5)	0.9 NS
min FeAA	1 ± 0.3 1 (0.3)	1 ± 0.2 1.1 (0.3)	1 ± 0.3 0.9 (0.4)	1 ± 0.3 1.1 (0.3)	0.8 NS
min MAC	0.5 ± 0.1 0.5 (0.2)	0.5 ± 1.1 0.6 (0.2)	0.5 ± 0.1 0.5 (0.2)	0.5 ± 0.2 0.5 (0.2)	0.5 NS
Stage 4—Post-Anesthesia Care Unit					
max HR (beats/min)	78.7 ± 15.9 75.3 (20)	78.2 ± 14.9 79.5 (19.8)	79.3 ± 18.4 75.3 (28.5)	78.6 ± 14.9 72.3 (13.5)	1.0 NS
max SAP (mmHg)	143.5 ± 23.4 142 (28.5)	139.2 ± 21.7 139 (25.8)	137.1 ± 24.6 137.7 (36)	155.1 ± 20.8 157.8 (29)	0.5 NS
max MAP (mmHg)	103.2 ± 14.5 105 (18.5)	101.9 ± 16.4 103.3 (24.5)	99.6 ± 13.7 101 (20.5)	108.5 ± 12.1 108.8 (13)	0.2 NS
max DAP (mmHg)	75.4 ± 11.9 75 (13)	73.6 ± 13.8 72.5 (16.5)	75 ± 11.4 75.5 (14)	77.6 ± 10.3 77.1 (12)	0.6 NS
max SPI	70.6 ± 12.8 71 (13)	67.2 ± 9.3 67 (14.5)	71.9 ± 17.2 74.3 (20)	73.2 ± 10.4 71.8 (11.3)	0.2 NS
min HR (beats/min)	64 ± 15 60 (18)	64.2 ± 11.8 62 (19.3)	60.7 ± 15.5 61 (23.5)	67.3 ± 17.5 59 (20)	0.4 NS
min SAP (mmHg)	119.2 ± 24.6 118 (32)	112.7 ± 20.9 114 (27)	109.4 ± 22 109 (29)	136.6 ± 22.6 137.5 (20.5)	RPV vs. MT, *p* = 0.01; BPV vs. MT, *p* = 0.002
min MAP (mmHg)	86.5 ± 14.6 86.5 (20)	84.2 ± 14.9 82.5 (17.5)	81.2 ± 13.6 80 (19)	94.6 ± 12.3 95 (14.5)	BPV vs. MT, *p* = 0.02
min DAP (mmHg)	61 ± 12.2 63.7 (18.5)	55.5 ± 12.8 51.3 (18.5)	60 ± 10.7 59 (13.5)	68.1 ± 9.9 67.5 (6.8)	RPV vs. MT, *p* = 0.006
min SPI	44.2 ± 14.1 43 (17)	42.5 ± 10.3 43 (13.5)	39.6 ± 15.6 41.3 (20)	50.8 ± 14.4 50.8 (24)	0.08 NS

RPV—ropivacaine; BPV—bupivacaine; MT—metamizole/tramadol; HR—heart rate; SAP—systolic arterial pressure; MAP—mean arterial pressure; DAP—diastolic arterial pressure; SE—state entropy; SPI—surgical pleth index; FiAA—fraction of inspired sevoflurane; FeAA—fraction of expired sevoflurane; MAC—minimal alveolar concentration of sevoflurane; OLIAAR—open lumbar infrarenal aortic aneurysm repair; SD—standard deviation; Me—median; IQR—interquartile range; NS—not significant.

## Data Availability

The original contributions presented in this study are included in the article/supplementary material; further inquiries can be directed to the corresponding author.

## Supplementary Materials

The following supporting information can be downloaded at https://www.mdpi.com/article/10.3390/ph17111497/s1, Figure S1: First postoperative pain perception depending on the group allocation; Figure S2: Mean values of min SAP, MAP, and DAP depending on the group allocation during the OLIAAR surgery.
