# Supplementary material for: Adequacy of Anesthesia Guidance for Combined General/Epidural Anesthesia in Patients Undergoing Open Abdominal Infrarenal Aortic Aneurysm Repair; Preliminary Report on Hemodynamic Stability and Pain Perception
Source: Pharmaceuticals (Basel). 2024 Nov 7;17(11):1497. doi: 10.3390/ph17111497 (PMC11597749; doi:10.3390/ph17111497)
Supplement: Supplementary file 1 [file pharmaceuticals-17-01497-s001.zip › pharmaceuticals-3249524-supplementary.pdf]

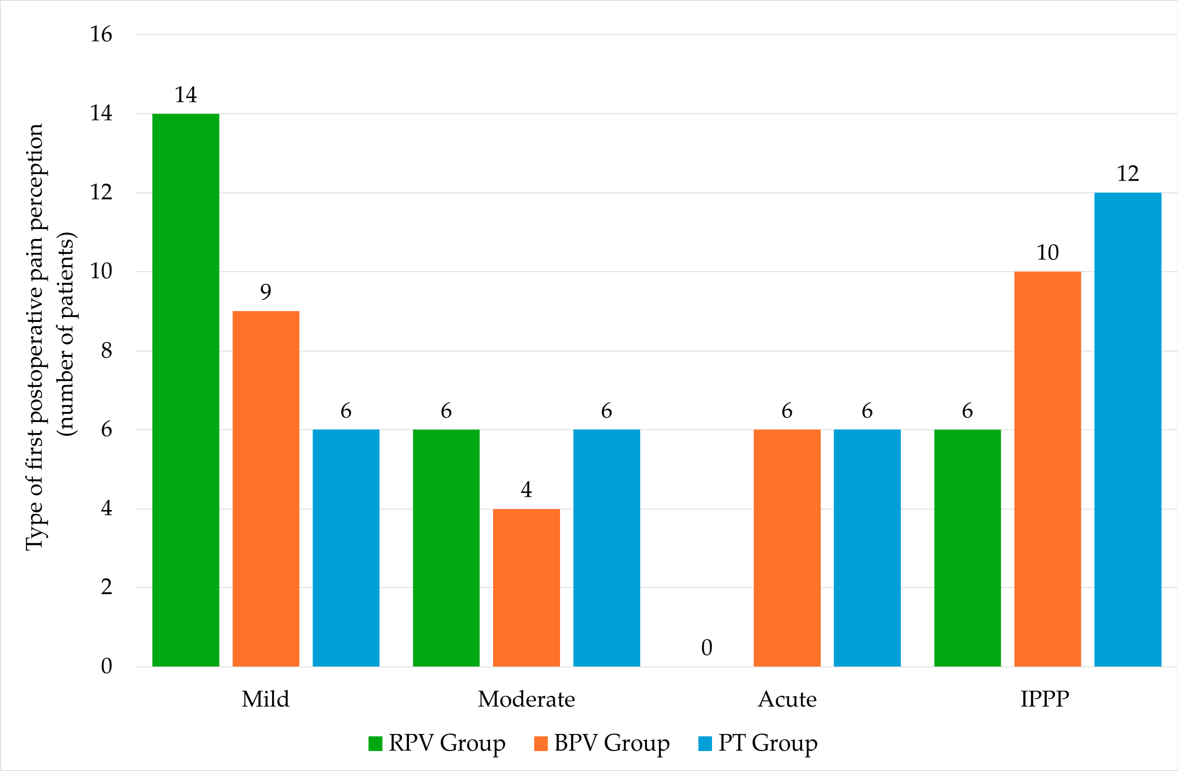

Supplementary Figure S1. First postoperative pain perception depending on the group allocation.

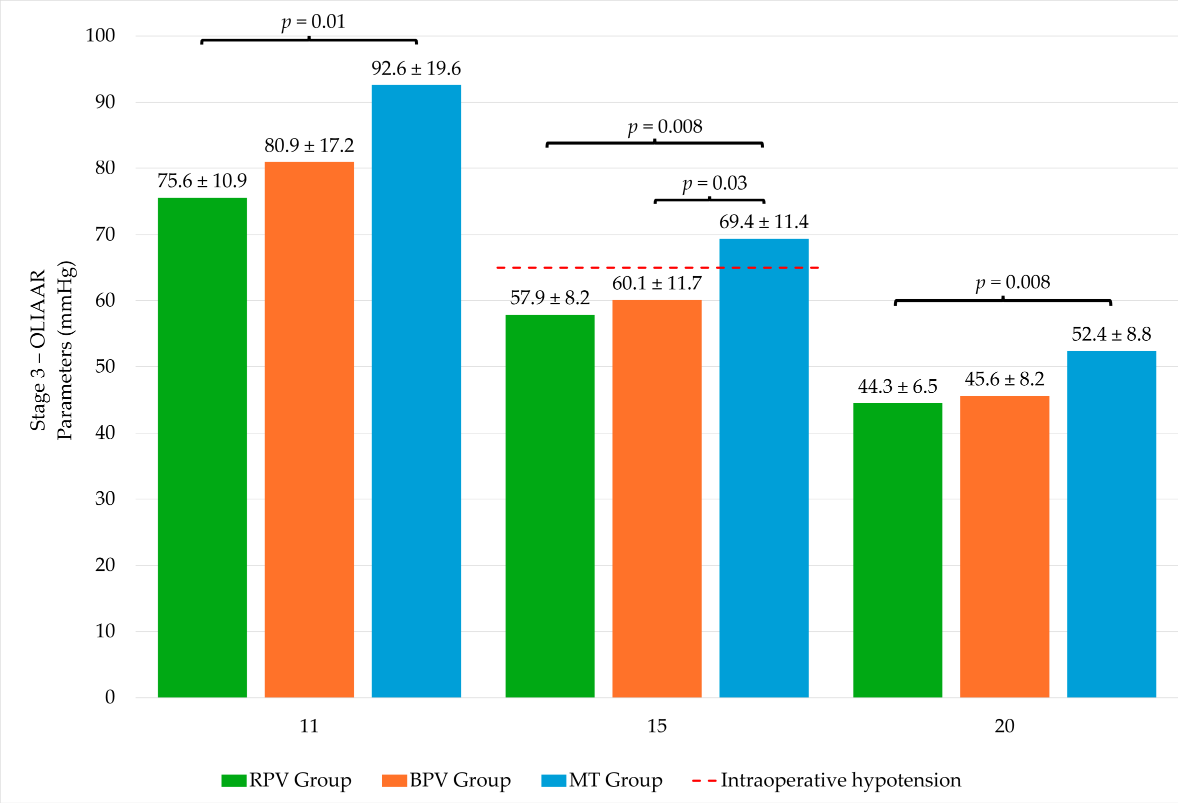

Supplementary Figure S2. Mean values of min SAP, MAP, and DAP depending on the group allocation during the OLIAAR surgery.
